# Estimation of hourly average illuminance under clear sky conditions in Chongqing

**DOI:** 10.1371/journal.pone.0237971

**Published:** 2020-08-24

**Authors:** Ying He, Xinshuo Zhang, Li Quan

**Affiliations:** 1 Faculty of Architecture and Urban Planning, Chongqing University, Chongqing, China; 2 Key Laboratory of New Technology for Construction of Cites in Mountain Area, Chongqing University, Chongqing, China; 3 Faculty of Chongqing Jianzhu College, Chongqing, China; Universidade de Vigo, SPAIN

## Abstract

Satellite-based methods are proposed for the estimation of clear day average hourly illuminance from satellite data under local climate conditions. First, aerosol optical depth (AOD) data collected using a ground-based sun photometer were used to calibrate the satellite remote sensing AOD data. Next, we screened for the factors affecting the illuminance of clear sky and detected three important factors, namely the sine of the solar altitude angle, aerosol optical thickness, and atmospheric precise water content. Finally, based on the AOD data of satellite remote sensing, combined with the local illumination data and meteorological data, a clear sky average hourly illumination model in Chongqing was established via the regression method. There was good agreement between the calculated and the measured values of clear day average hourly illuminance, with a root mean square difference and mean bias difference of 22% and -0.05%, respectively. The model was used to map clear day annual, quarterly, and monthly average hourly illuminance. The maps show the clear day annual, seasonal, and monthly variations of average hourly illuminance in Chongqing.

## 1. Introduction

The utilization of daylight for illuminating building interiors can bring about significant savings in terms of building electricity consumption [1]. For this reason, daylight-integrated buildings and daylight equipment have been developed in many countries [2,3]. Different climate conditions in different regions lead to different daylighting environments. Therefore, according to the daylight climate conditions, some countries have divided the area into daylight climate zones and formulated corresponding daylight strategies. In China, daylight climate zones are divided into five categories [4].

Daylight consists of direct and diffuse sky light. Daylight climate can be divided into three sky conditions, namely clear days, partly cloudy days, and overcast days. The use of diffuse sky lights is preferable for illuminating building interiors under overcast conditions. As a result, the amount of diffuse illuminance available at a location is generally required for assessing the potential use of daylight at that location, which is accomplished with metrics such as the daylight factor. Recently, dynamic daylight performance metrics were proposed by Mardaljevic [5]. The key advantage of dynamic daylight performance metrics compared to static metrics is that these dynamics ones consider the quantity and character of daily and seasonal variations of daylight for a given building site together with irregular meteorological events [6]. So clear days and partly cloudy days become important parts of dynamic daylight performance. Ideally, illuminance data should be obtained from a dense network of daylight stations. However, because of cost considerations, there are very few daylight stations around the world. A number of authors have proposed luminous efficacy models for estimating the illuminance from irradiance [7,8]; however, measurements of irradiance around the world are also limited.

As illuminance is part of broad-band solar radiation, which is derivable from satellite data [9,10], it is possible to estimate the illuminance from satellite data. The advantage of this satellite technique is that illuminance data can be obtained for all areas corresponding to satellite pixels. The first attempt in estimating illuminance from satellite data was conducted in the SATEL_LIGHT project [11]. Later on, S. Janjai’s research group proposed a physical approach for calculating the global illuminance from satellite data [12] and displayed the results as illuminance maps. A similar approach has been proposed by He and Ng [13]. The above satellite remote sensing methods use the image pixel gray value to obtain the cloud index, and then, the ground illumination is obtained.

Natural illumination that reaches the ground is influenced by clouds, atmospheric conditions (including the basic atmospheric composition, aerosols, and water vapor), and changes in solar radiation. The major factors are cloud cover and aerosols [14]. The impact of clouds on natural light is quite complex. Different heights, types, and thicknesses, as well as different physical, chemical, and optical properties of clouds, will produce different effects on daylight illumination. Aerosols are another important and complex influencing factor of natural light. An increase in the aerosol content increases the atmospheric optical thickness, and this correspondingly reduces the daylight illumination that reaches the surface. Aerosol optical depth (AOD) is the most commonly used and most important aerosol research parameter. The AOD describes the aerosol’s attenuation of light, and it is defined as the vertical integration of aerosols in the atmosphere. There two kinds of measurement methods for the AOD, namely, the ground-based remote sensing method [15] and satellite remote sensing method [16,17].

Clear day illuminance is an important part of daylight climate. As clear days are cloudless, these days represent useful targets for further research. The objective of this work was to develop a method for clear day illuminance estimation from satellite AOD and meteorological data. The study area of the work was focused on Chongqing municipality.

## 2. Materials and methods

The proposed method is divided into two steps. The first step is to derive the AOD from satellite data; then, the satellite AOD data is calibrated using ground-based AOD test data. The second step is to establish a clear day illuminance model by using the multiple regression technique based on average hourly illuminance data from ground tests on clear days and satellite AOD data, in combination with local meteorological parameters.

### 2.1 Site description

**Fig 1:**
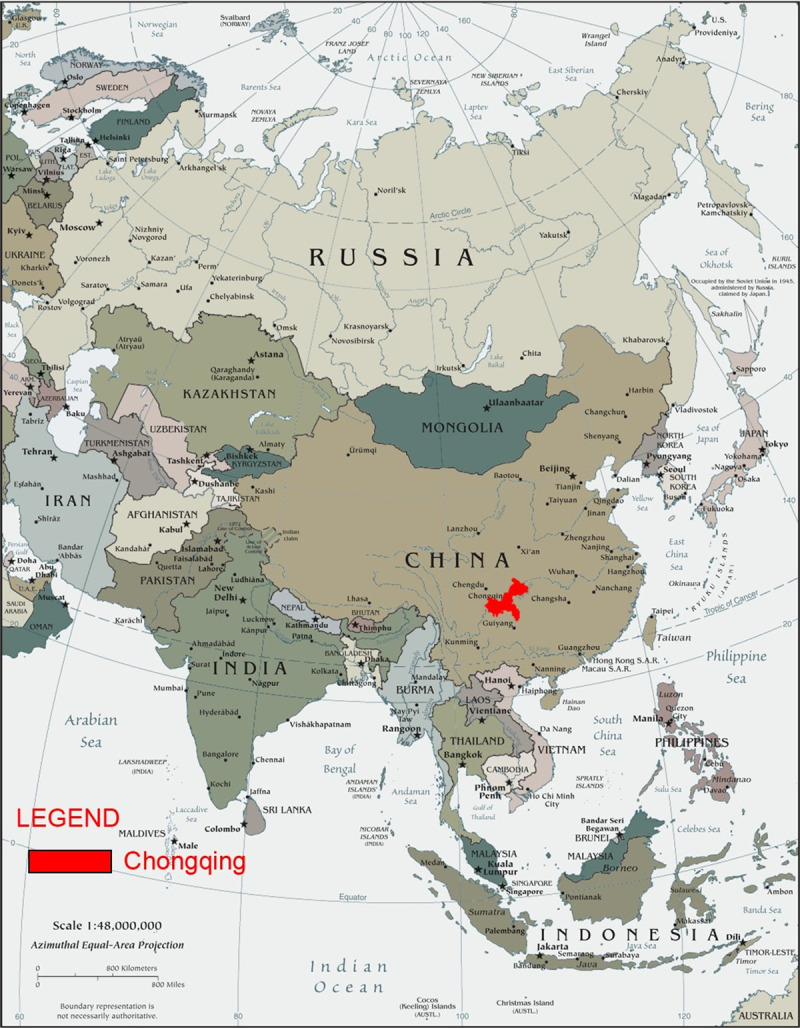
Chongqing’s geographical position in Asia.

Chongqing municipality is located in the subtropical inland area of the Northern Hemisphere. Chongqing is situated in the southwestern part of China; the upper areas are located along the Yangtze River, the eastern edge falls along the Sichuan Basin, which is surrounded by mountains (Fig 1). With a total area of 82,400 km^2^, the municipality includes 38 districts or counties. Chongqing is a region of stable meteorological conditions, and there are typically low winds and high relative humidity levels in the atmosphere. Chongqing also contains urban industrial areas, and thus, irregular transported dust was present throughout the whole studying period.

### 2.2 Instrument and data

#### 2.2.1 AOD measurements

**Fig 2:**
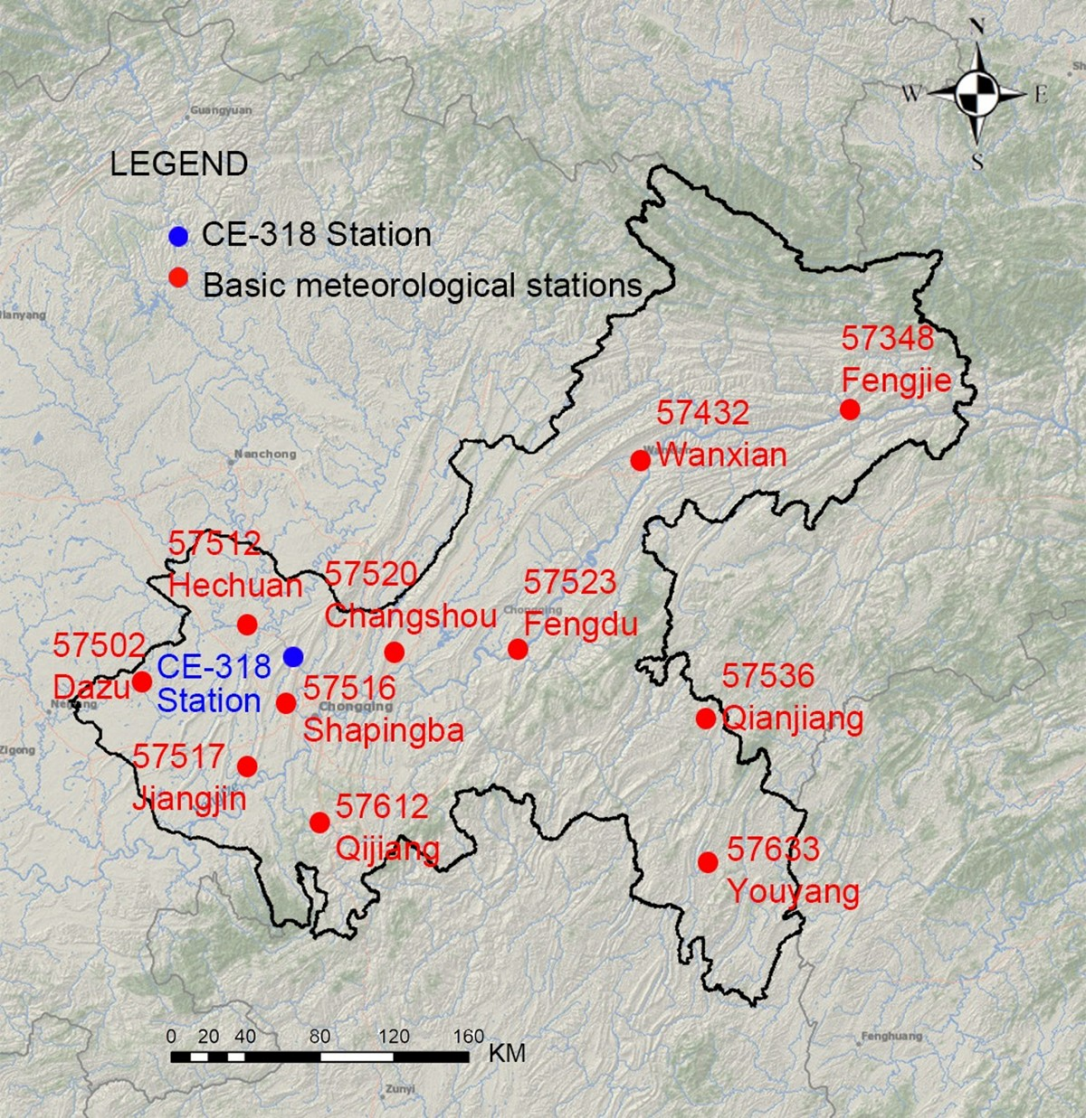
Eleven benchmark and basic meteorological stations and the CE-318 station in Chongqing.

Aerosol optical properties were continuously observed using a CE318 sun-photometer (Cimel Electronique, France) placed on the roof of the Fenghuang Building (106.51°E, 29.62°N), which is located in the central urban area of Chongqing (Fig 2). This instrument is a high-precision multiband photometer that measures the optical properties of the atmosphere through measurements of the direct sun irradiance and diffuse sky radiance, and it allows for the quantification and physical-optical characterization of the atmospheric aerosols [18]. The standard measuring schedule used with the CE-318 instrument was composed of direct sun triplets every 15 min and sky diffuse almucantar and principle plane scenarios every 1 h.

The data collected by the CE-318 instrument were AOD data values on sunny days. Because there are many rainy days in summer and winter in Chongqing, the number of sunny days are different each month; thus, the number of effective observation data points obtained in different months were different in this study. The CE-318 AOD test data had high accuracy; thus, these data were used as criterion values to calibrate the satellite AOD data. The whole year of AOD observation data from 2017 was used in this study.

#### 2.2.2 Illuminance measurements

To formulate a satellite-based clear day hourly illuminance model and conduct the validation of the method, it was necessary to have ground-based clear day hourly illuminance data. We used a daylight climate station located in the Chongqing University Campus B (106.46°E, 29.57°N), which is adjacent to the Shapingba observation station (Fig 2). At the station, global illuminance and diffuse illuminance were measured at 10 min intervals from June 2016. Combined with the meteorological data, global illuminances were partitioned into the following two data sets: clear day illuminances and other day illuminances. The clear day average hourly illuminances in 2017 were obtained and used in this work. The data set was further screened to match the AOD data and used for fitting the models, and other data were combined with the ground AOD data to evaluate the model accuracy.

#### 2.2.3 Meteorological data

The meteorological data were obtained from the Dataset (V3.0) of Daily Values of Climate Data of China Meteorological Data Service Center (http://data.cma.cn/search/keywords.html), which contains daily data on the air pressure, temperature, precipitation, evaporation, relative humidity, wind direction, wind speed, sunshine hours, and 0 cm ground temperature elements from January 1951 onward that were collected at 699 benchmark and basic meteorological stations. There are 11 benchmark and basic meteorological stations in Chongqing (Fig 2), which include 57348 Fengjie, 57432 Wanxian, 57502 Dazu, 57512 Hechuan, 57516 Shapingba, 57517 Jiangjin, 57520 Changshou, 57523 Fengdu, 57536 Qianjiang, 57612 Qijiang, and 57633 Youyang. The Shapingba station is located in the central districts of Chongqing.

### 2.3 Satellite AOD data acquisition and correction

#### 2.3.1 Satellite AOD data acquisition

The Moderate Resolution Imaging Spectroradiometer (MODIS) is a key instrument onboard the Terra and Aqua satellites launched by the National Aeronautics and Space Administration (NASA), and it acquires data in 36 spectral bands and views the Earth twice in the morning and afternoon [19]. The true color images used to identify aerosols were retrieved from MODIS. Recently, the newest MODIS Collection 6.1 (C6.1) AOD products have become available with various refinements and improvements made to both the radiation calibration and Dark Target (DT) and Deep Blue (DB) algorithms [20]. Related images can be downloaded from the following website: https://worldview.earthdata.nasa.gov/. In this study, a total of 365 scene data in 2017 were obtained.

#### 2.3.2 Spatio-temporal matching of ground-based and satellite AOD data

The ground AOD data are based on the continuous observation of a point, and the satellite AOD data represent the regional AOD data during the transit of the satellite.

To compare the ground data with the satellite data, the two kinds of data had to be at the same spatial and temporal scale. In this study, the principle of spatial matching was used to set the ground observation station as the center and average the grid data within a 30 km circumference, for which the corresponding AOD data were obtained. Time matching involved matching based on the standards of satellite transit times and the station observation times within 30 min.

The CE318 AOD includes data from the 340 nm, 380 nm, 440 nm, 500 nm, 670 nm, 870 nm, and 1020 nm wave bands. The center lengths of the MODIS AOD product wave band were 470 nm, 550 nm, and 650 nm, so the ground data were inconsistent with the satellite data wave bands. The wave band of 550 nm is the most commonly used to study the AOD [21]. Therefore, it was necessary to interpolate the wavelength of the CE318 data to obtain 550 nm data, and then, data were matched. Wavelength interpolation can be obtained by the Angstrom relation given as follows:

$$\tau (\lambda )=\beta ∙\lambda^{-\alpha }$$ (1)

where τ(λ) is the AOD at wavelength λ, *α* is the wavelength index of the Angstrom, and β is the atmospheric turbidity coefficient.

The preprocessed MODIS AOD data and CE-318 AOD data were matched in time and space, and 71 sets of AOD data were extracted.

#### 2.3.3 Satellite AOD data correction

**Fig 3:**
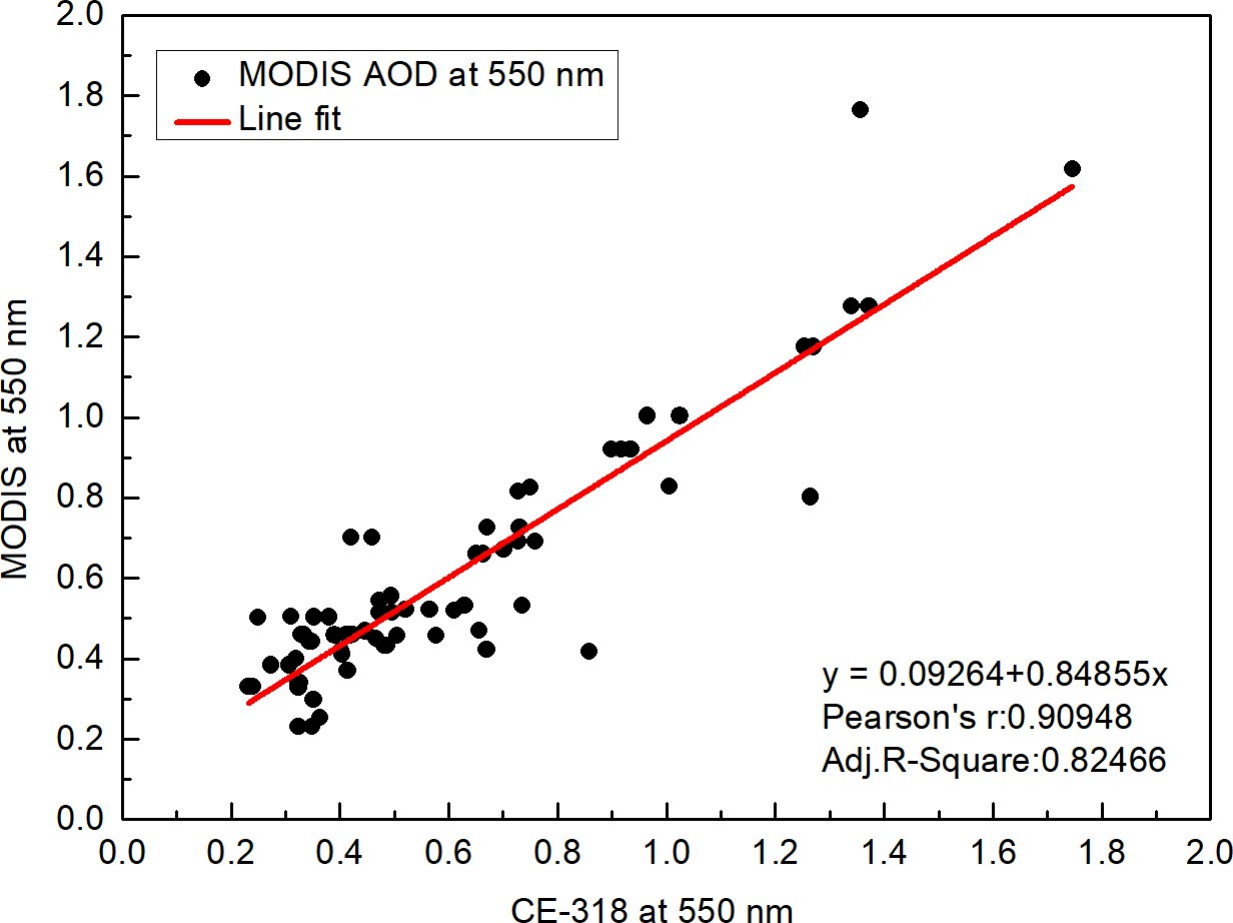
Comparison between MODIS AOD products and CE-318 AOD products.

Based on the correlation analysis of the data extracted from the spatiotemporal matching, a scatter plot was obtained by taking the 550 nm CE-318 AOD data as the abscissa and the 550 nm MODIS AOD data as the ordinate. The linear regression between the two sets of variables is shown in Fig 3, and the best fitted equation is as follows:

$$\tau_{M}=0.09264+0.84855\tau_{G}$$ (2)

where *τ*_*M*_ is the MODIS AOD and *τ*_*G*_ is the CE-318 AOD.

Fig 3 shows a correlation coefficient (R) of 0.909 and root mean square difference (RMSD) of 0.82. Hence, MODIS AOD products in Chongqing have high inversion accuracy, and these can be used in AOD-clear day illumination model research.

### 2.4 Development of a clear day hourly illuminance model

#### 2.4.1 Correction factor screening

Sunlight is influenced by many factors in the process of atmospheric transmission. Different illuminance and radiation models have been put forward on the basis of different meteorological parameters. The clear day illuminance value is affected by the geographic latitude, altitude, absolute humidity, and sunshine hours [22]. The 6S model (Second Simulation of the Satellite Signal in the Solar Spectrum) [23] has indicated that the illuminance reaching the ground on a clear day is related to the cosine value of the solar zenith angle and the atmospheric optical depth. The surface solar radiation model uses variables such as Rayleigh scattering, water vapor absorption, aerosol absorption, and scattering to parameterize the model. According to a model sensitivity study, the zenith angle, precipitable water amount, and AOD are the most important factors that affect the ground radiation [24].

Based on the above research, the clear day illuminance model in this study screened out aerosol optical depth τ and employed the following three sets of correction factors: solar altitude angle h/solar altitude angle sine value Sinh, relative humidity Rh/atmospheric precipitable water content w, and average pressure P.

#### 2.4.2 Data preprocessing

First, the modeling data had to be matched in terms of the temporal and spatial scales. Because the site location of the meteorological data used in this modeling was very close to the illuminance data collection point, it was regarded as one point approximately, and the geographic coordinates of the meteorological site were used to match the AOD data. Take the location of meteorological monitoring station as the center, read the data within a 30 km circumference around the station every day and average, and then compute the daily AOD data. The time above the transit stations of Terra and Aqua is from 10:00 to 15:00 every day. For the illuminance data, the data acquisition interval was 10 min; thus, we added up and averaged the 10 min data, and then, data were converted to hourly data. Next, the data points closest to the AOD observation time every day were taken as the matching data. The average daily meteorological data of 2017 were used to match the AOD data.

The solar altitude angle *h* can be calculated by the latitude and longitude of the measuring point and the true solar time. Relative humidity *Rh* and average pressure *P* can be found within the Dataset (V3.0) of Daily Values of Climate Data of China Meteorological Data Service Center. Atmospheric precipitable water content *w* can be expressed by the dew point temperature *t*_*d*_ on the ground.

$$w=10^{0.033t_{d}-0.151}$$ (3)

$$t_{d}=\frac{237.7\gamma (t,Rh)}{17.27-\gamma (t,Rh)}$$ (4)

$$\gamma (t,Rh)=\frac{17.27t}{237.7+t}+\ln\left(Rh∕100\right)$$ (5)

In the above equations, the unit of temperature *t* and dew point *t*_*d*_ is in centigrade, and the relative humidity *Rh* is expressed as a percentage.

After removing the null value and mismatching value, 158 sets of data were obtained for the clear sky illuminance model.

#### 2.4.3 Model formulation

In this study, a multiple linear regression model was used for modeling. The dependent variable was illuminance, and the six independent variables preliminarily selected were as follows: aerosol optical depth τ, solar altitude angle sine *Sinh*, solar altitude angle *h*, relative humidity *Rh*, atmospheric precipitable water content *w*, and average air pressure *P*. *Sinh* and *h*, *Rh* and *w* are independent variables with similar physical significance. In the modeling, we explored which independent variables were the most suitable for this model.

In this study, we use the forward method to screen the independent variables. The forward method uses a standard for selecting the independent variables in advance. First, there are only constant terms in the equation without independent variables. Then, according to the contribution of independent variables to the dependent variables, the equation is selected in order from large to small. Whenever a new independent variable enters the equation, the contribution of each variable outside the equation (after deducting the influence of the selected variable) to the dependent variable is recalculated. Repeat the above steps until the independent variables out of the equation do not meet the inclusion criteria and no independent variables can be introduced into the equation. This method only considers the selection of an independent variable according to the given standard, and once an independent variable enters the equation, it will not consider the elimination of the independent variable. The JASP (0.12.2.0) statistical program was used for this variable screening. JASP is an open-source statistics program that is free, friendly, and flexible. Developed by a dedicated team of programmers and academics, JASP aims to help people around the world draw informative conclusions from their data.

Before modeling, we needed to analyze the descriptive statistical information of each variable and the correlation between each variable, so as to judge whether the data met the requirements of modeling. The correlations among variables are summarized in Table 1; the correlation between illuminance and several independent variables was relatively evident, and there were certain correlations between independent variables, such as the air pressure, precipitable water content, and relative humidity. Therefore, when modeling, attention should be paid to excluding the influence of the correlation between independent variables on the model.

**Table 1:** Pearson correlation between the independent variables of the model.

	*Ec*	*τ*	*sinh*	*h*	*Rh*	*P*	*w*
*Ec*	1.000						
*τ*	-0.482	1.000					
*sinh*	0.893	-0.413	1.000				
*h*	0.879	-0.379	0.988	1.000			
*Rh*	-0.635	0.475	-0.677	-0.675	1.000		
*P*	-0.769	0.319	-0.846	-0.831	0.676	1.000	
*w*	0.756	-0.291	0.773	0.750	-0.490	-0.892	1.000

Based on all matching data in 2017, the JASP (0.12.2.0) “linear regression” module was used for modeling. For the dependent variable, “illuminance” was selected, and for the independent variables, “*τ*,” “*Sinh*,” “*h*,” “*Rh*,” “*w*,” and “*P*” were selected. The selection method of the independent variables was set to “stepwise,” and the selection and elimination criteria were set to P ≤ 0.05 and P ≥ 0.1 according to the P value.

Table 2 lists the screening process for the model. Model 1 was constant. Model 2 selected the variable “*Sinh*” to enter the equation, thus indicating that *Sinh* had the greatest contribution to the illumination. Model 3 selected the variable “τ” to enter the equation on the basis of model 2, and model 4 selected the variable “*w*” to enter the equation on the basis of model 3; then, the whole process of independent variable screening ended. These results indicated that the other variables did not meet the conditions of entering the equation, and the existing variables in the equation did not need to be eliminated. The contribution of average relative humidity *Rh* and average air pressure *P* to illuminance was small or replaced by other independent variables existing in the equation, so these terms did not enter the equation.

Table 2 also shows the relevant indicators of the fitting model. Model 2 contains *Sinh*, model 3 contains *Sinh* and τ, and model 4 contains *Sinh*, τ, and *w*. With the introduction of independent variables in turn, the number of complex phase relations R and the determination coefficient R^2^ of the model gradually increased. The final output R was 0.909 and the final R^2^ was 0.826, which indicate that the model had a good fit. The explanatory degree of the independent variables *Sinh*, τ, and w to the overall variation was 82.6%. The adjusted R^2^ also increased with the introduction of the independent variables, which indicates that the introduced independent variables had a practical explanatory effect on the dependent variables.

**Table 2:** Change of the coefficient of determination in the model.

Model	Variable	R	R^2^	Adjustment R^2^	R^2^ change	F change	p
1	constant	0.000	0.000	0.000	0.000	-	-
2	constant+sinh	0.893	0.798	0.797	0.798	617.244	< .000
3	constant+sinh+τ	0.902	0.814	0.811	0.016	12.960	< .000
4	constant+sinh+τ+*w*	0.909	0.826	0.822	0.012	10.511	0.001

After stepwise regression, the final model equation obtained was as follows:

$$E_{c}=\mathrm{asin}h+b\tau +cw+d$$ (6)

where *E*_*c*_ is clear day hourly illuminance, *h* is solar altitude angle, *τ* is aerosol optical depth, *w* is the atmospheric precipitable water content, which can be calculated by the relative humidity and temperature, and *a*,*b*,*c*,*d* are the coefficients of the equation. In Chongqing, the values are as shown in Table 3.

**Table 3:** Coefficient values.

Coefficients	a	b	c	d
Values	113715.532	-7.568	3340.354	-31493.914

## 3. Results and discussion

### 3.1 Performance of the method

The method described in the preceding section is now ready for use to calculate the direct illuminance at all pixels of the satellite data. Although the method was logically developed, it was necessary to test its performance prior to `utilization. Because MODIS transits only once a day, and AOD data are affected by clouds, the number of data points was limited. To verify the validity of the model, we used the CE 318 AOD data in 2017.

**Fig 4:**
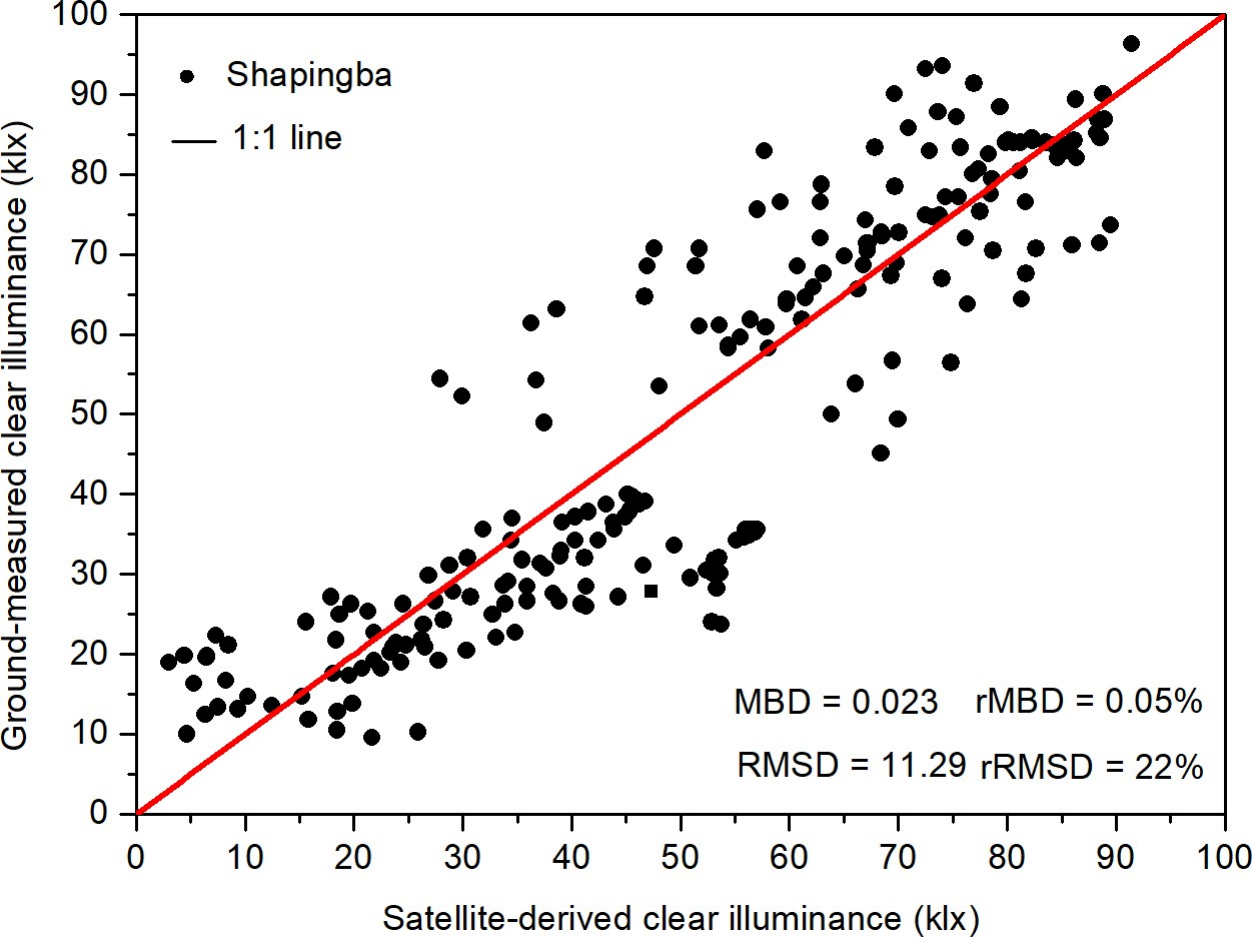
Measured and modeled clear day hourly illuminance at Chongqing in 2017.

Measured and modeled clear day hourly illuminances were compared (Fig 4) at the Chongqing University (Campus B) daylight climate observation station in 2017. Notably, the data points were scattered around the 1:1 line. The root mean square difference (RMSD) and mean bias difference (MBD) between the calculated and the measured clear illuminance are listed in Table 4. These resulted indicated the two data sets were in good agreement.

**Table 4:** MBD and RMSD of the clear model.

	MBD (klx)	rMBD	RMSD (klx)	rRMSD
Proposed model	0.023	0.05%	11.29	22%

### 3.2 Error analysis

From Fig 4, we can see the comparison between the model’s inverse illuminance and the measured illuminance. The model’s inverse illuminance better represented the actual illuminance, but there was still an error between the inverse illuminance and the actual illuminance. The possible sources of error in the model are discussed below.

First, the atmospheric precipitation content *w* used in the model uses daily averages rather than instantaneous values. The values may vary drastically during the day, so this will cause errors in the model inversion to a certain extent. Second, statistical analyses of the three independent variables used can explain 82.6% of the change in illuminance, but other factors that may have had an influence on the contrast were not reflected in the model, such as atmospheric molecular absorption and scattering and the effects of thin clouds. Finally, because the model was built using only one year of data, the data may have had abnormal extreme values, and this may have had a certain impact on the accuracy of the model.

### 3.3 Clear day hourly illumination inversion

Because MODIS satellite transit satellites are generally concentrated between 10:30 am and 2:30 pm, the data of clear sky illuminance retrieved by MODIS satellite can be used to reflect the daylight climate conditions in the noon period of clear days. The retrieval of MODIS satellite clear day illuminance was as follows.

First, based on the coordinates of the 11 meteorological stations in the Chongqing area (Fig 2), and by taking the coordinates of the surface meteorological stations as the center, the grid data of the surrounding 30 km range was read and averaged to obtain the AOD data of the 11 stations. The daily MODIS AOD data was read cyclically to obtain the whole year of 2017. We calculated the solar height angle *h* and *sinh* at that time based on the obtained MODIS AOD data time. Then, we used the daily average temperature and daily relative humidity obtained from the China Meteorological Data Network to calculate the atmospheric precipitation content *w*. Finally, the above parameters were input into Eq (3) to calculate the 2017 clear day average hourly illuminance values of 11 stations in Chongqing.

**Fig 5:**
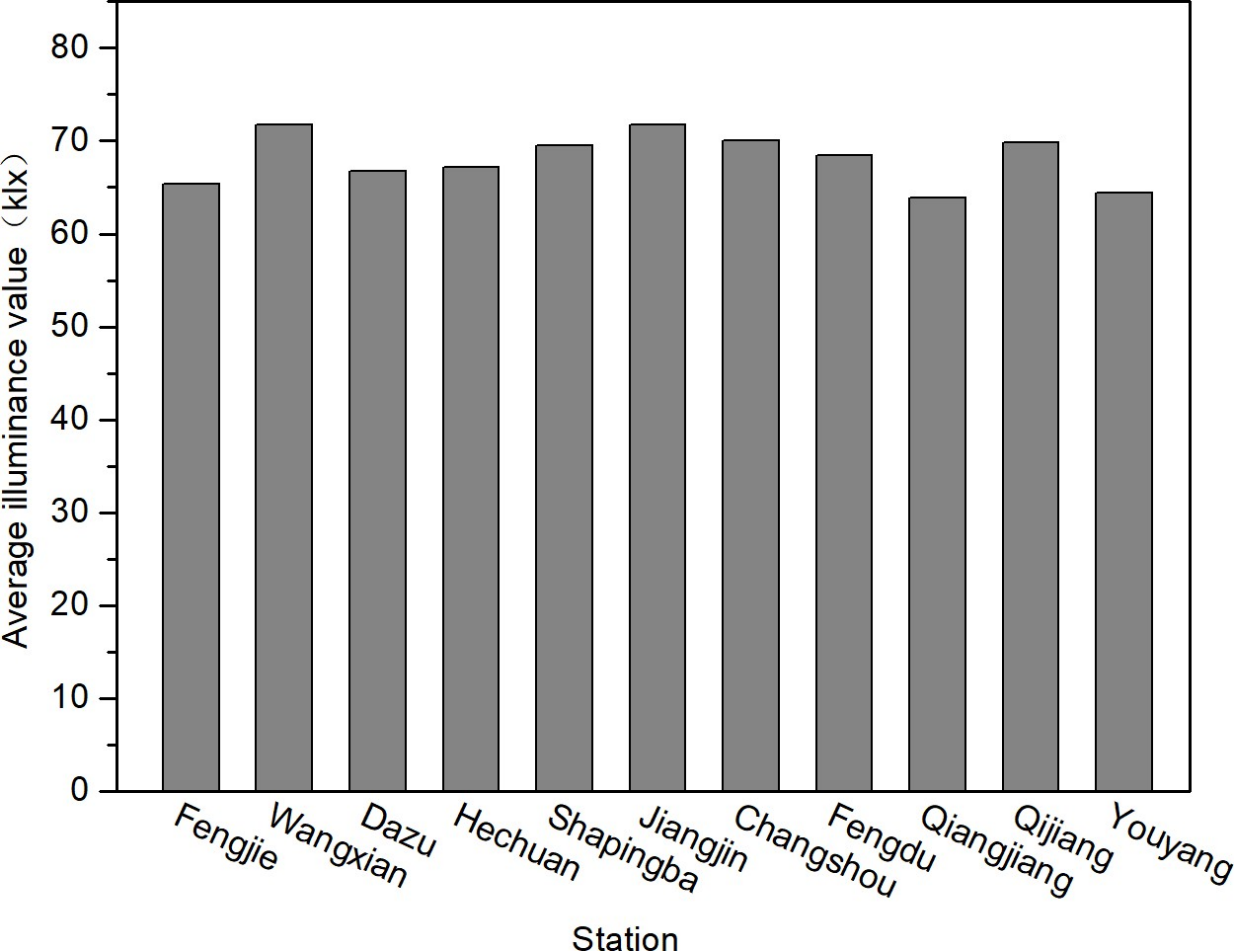
Chongqing clear day annual average hourly illumination values of 11 stations in 2017.

**Fig 6:**
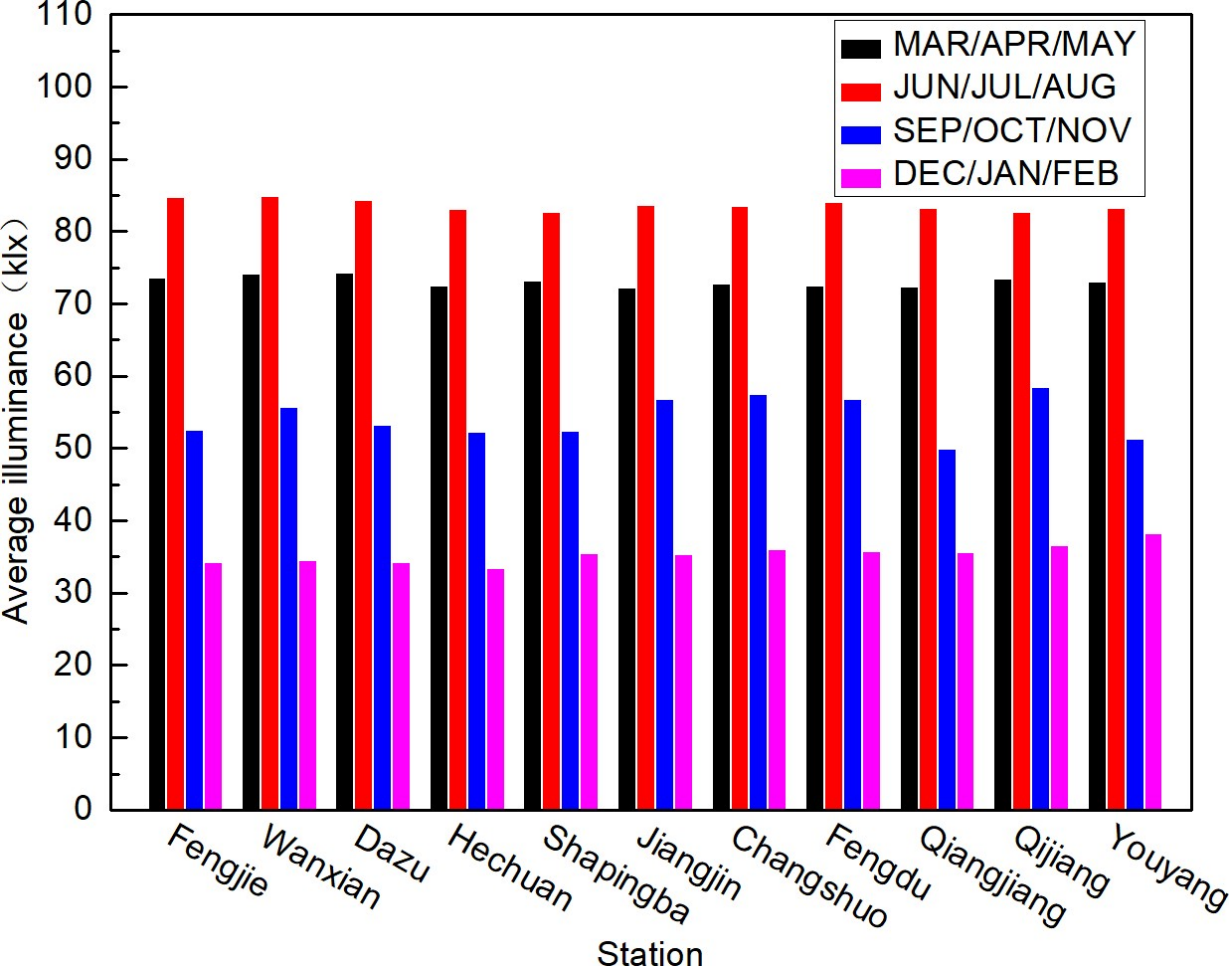
Chongqing quarterly clear day average hourly illumination values of 11 stations in 2017.

**Fig 7:**
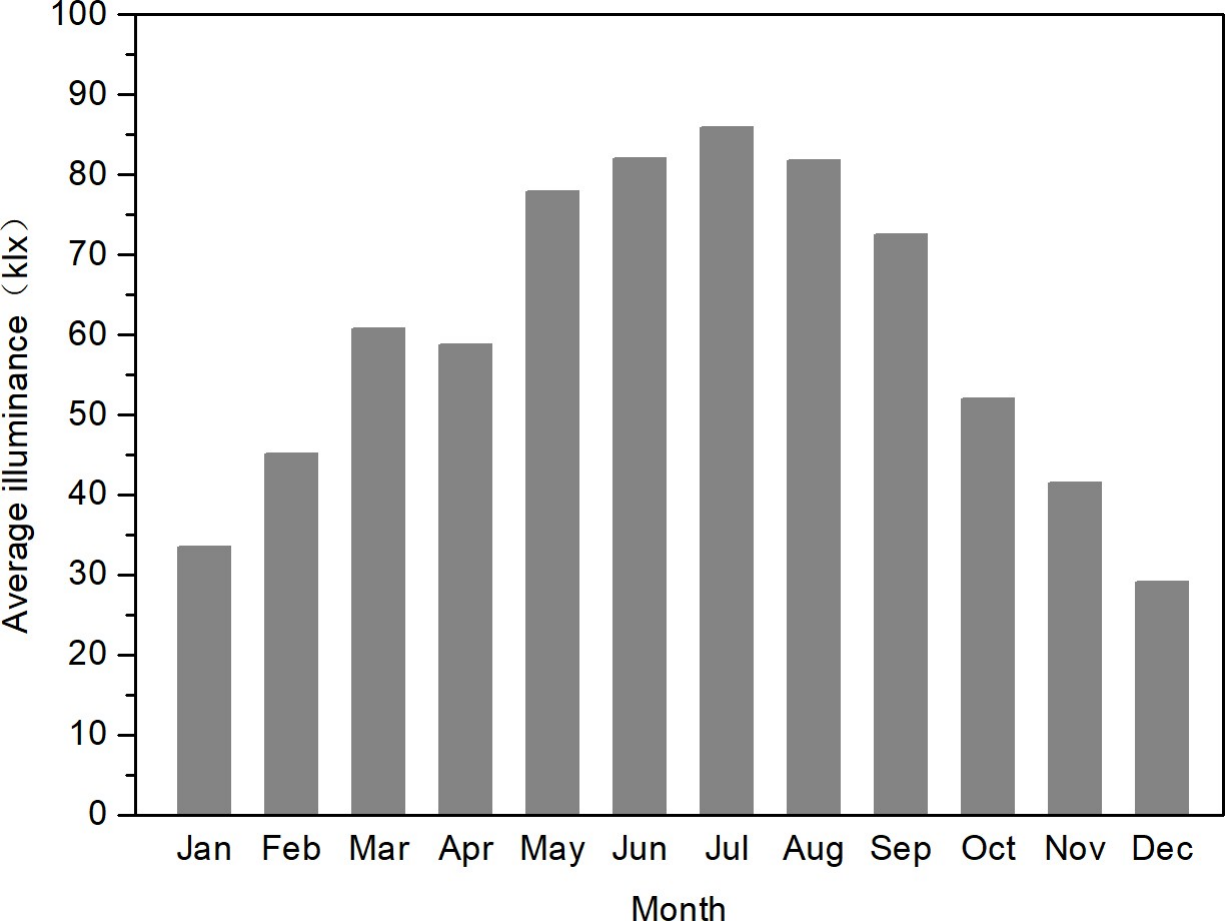
Chongqing clear day monthly average hourly illumination values of 11 stations in 2017.

To facilitate the analysis of the trends in illumination changes throughout the year, the annual average hourly illumination values (Fig 5), the quarterly average hourly illumination values (Fig 6), and the monthly average hourly illumination values (Fig 7) of 11 stations at noon were calculated. According to Chongqing meteorological conditions, December, January, and February represent winter, March, April, and May represent summer, June, July, and August represent autumn, and September, October, and November represent winter. The average annual hourly illuminance value was calculated by adding the annual clear day hourly illuminance value and dividing by the annual number of clear days; the quarterly average illuminance value and monthly average value were calculated in the same way as the annual average illuminance value.

Fig 5 shows that the difference among the clear day annual hourly illuminance values of each station in Chongqing area was similar, and the maximum difference with the mean value was 6.2%. Therefore, it can be concluded that the clear day annual hourly illuminance values were basically equal in Chongqing.

Fig 6 shows that the clear day hourly illuminances in Chongqing showed a trend of summer > spring > autumn > winter in terms of the temporal distribution, and the seasonal difference was very significant.

Fig 7 shows the change trend of clear day monthly average hourly illuminances in Chongqing throughout the year. From January to December, the illuminance of the clear sky rose first and then decreased. The results indicated that the illuminance values were large in summer and much smaller in winter.

### 3.4 Mapping clear day hourly illuminance

The AOD *τ* of the illuminance inversion model parameters can be obtained from satellite data, and the solar altitude angle *h* can be calculated according to the geographic latitude and time, but atmospheric precipitable water content *w* needs to be obtained by ground observations, so the illuminance distribution map of Chongqing was obtained by spatial interpolations. The digital map come from the National Catalogue Service For Geographic Information (www.webmap.cn).

**Fig 8:**
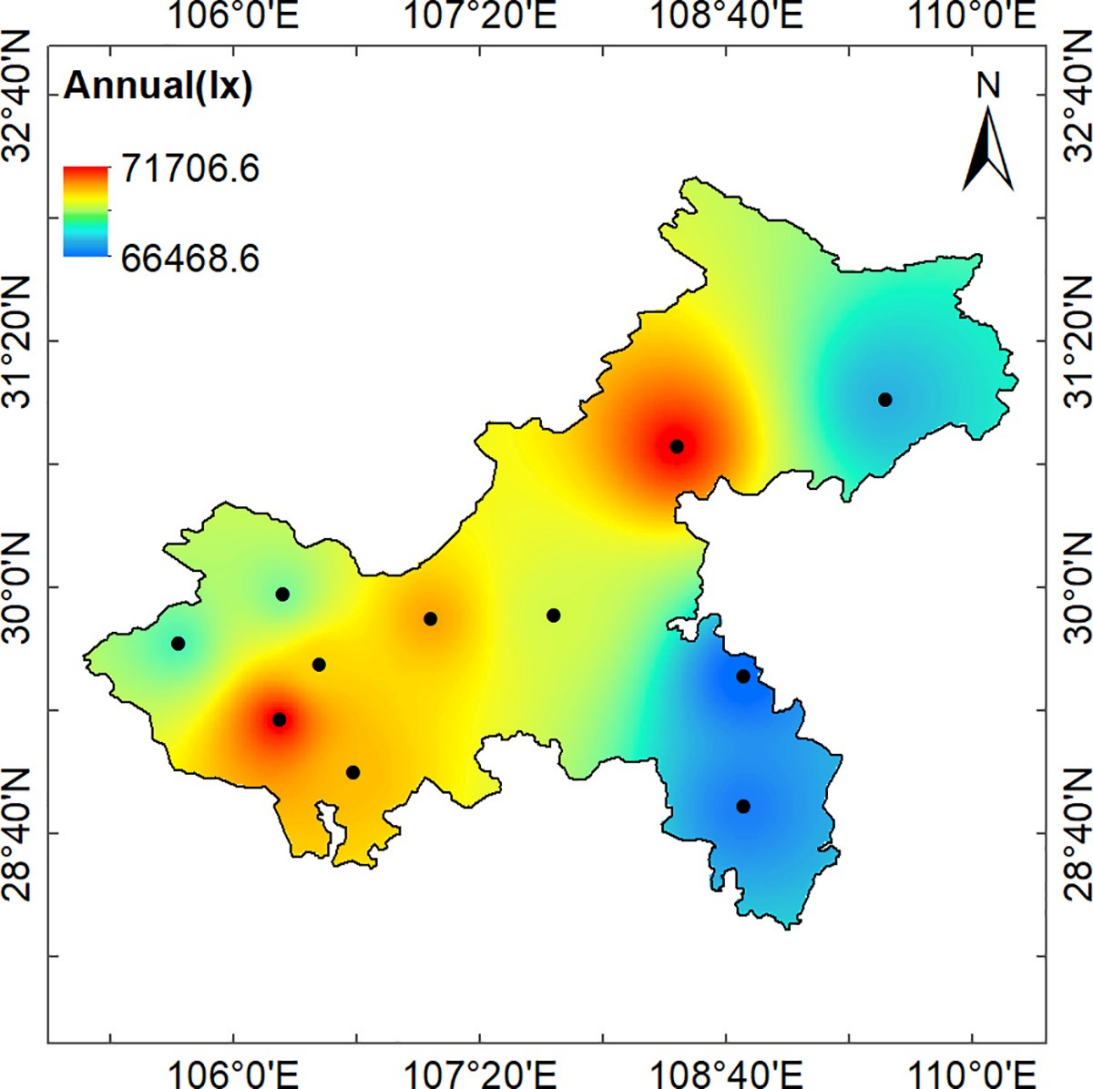
Chongqing annual average hourly illumination distribution at noon.

**Fig 9:**
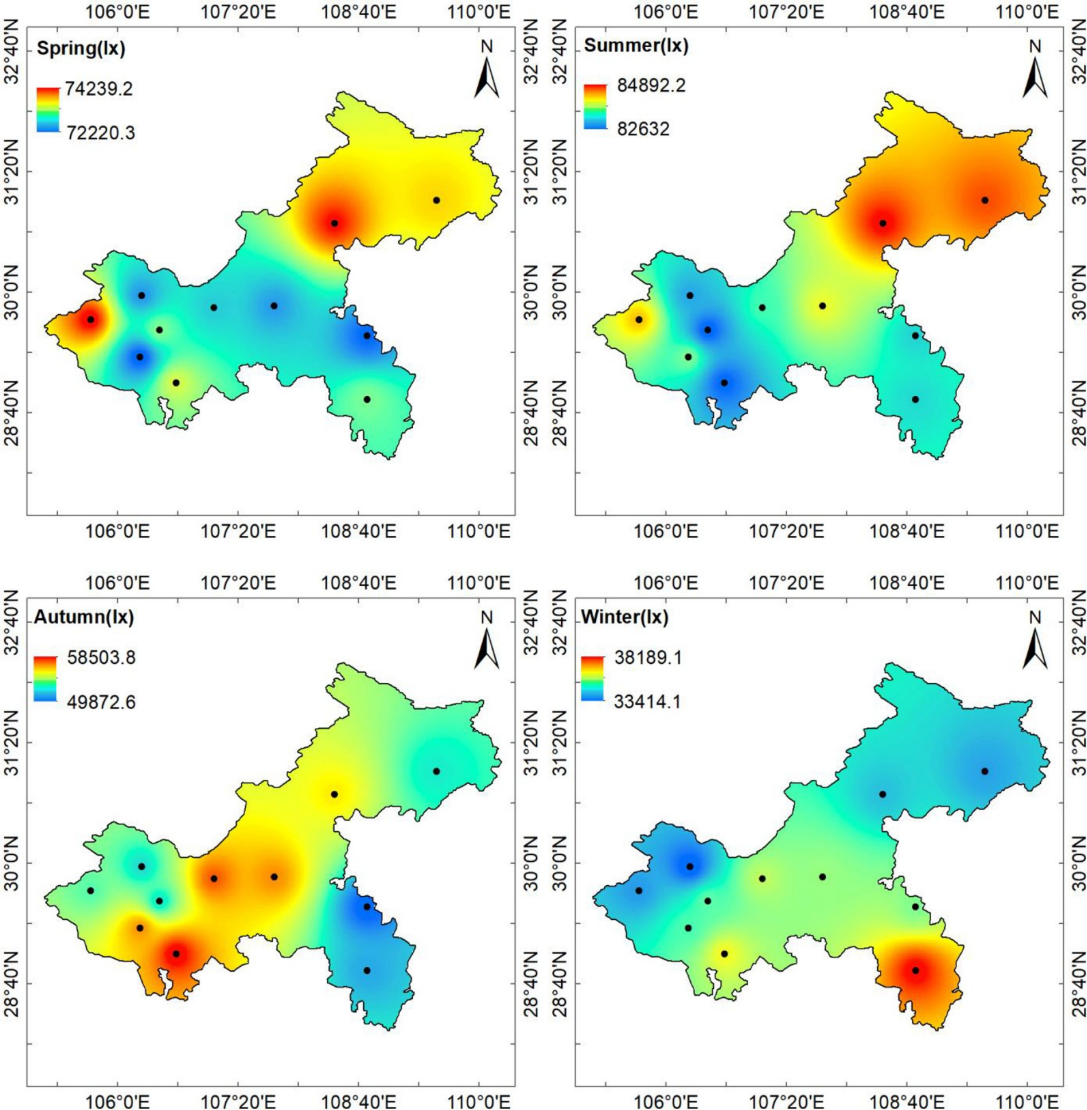
Chongqing quarterly average hourly illumination distribution at noon.

**Fig 10:**
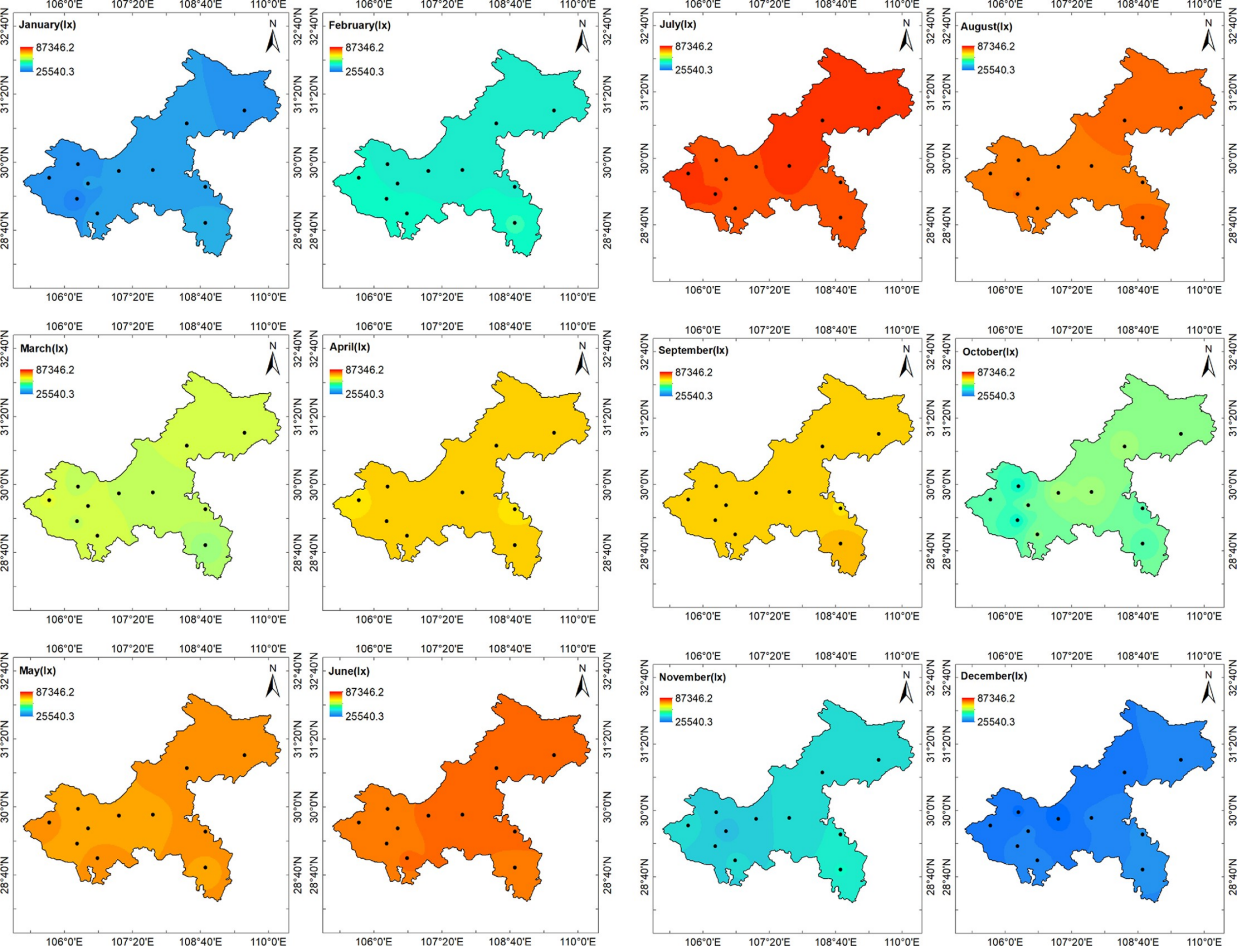
(a) Chongqing monthly average hourly illumination distribution with same color scale at noon (from January to June). (b) Chongqing monthly average hourly illuminance distribution with the same color scale at noon (from July to December).

Those results are displayed as maps showing the geographical distribution over Chongqing city of clear day annual average hourly illuminance values (Fig 8), quarterly average hourly illuminance values (Fig 9), and monthly average hourly illuminance values (Fig 10).

Fig 8 shows that the high value of the annual average hourly illuminance occurred in the Wanxian District and Jiangjin District, while the annual average illuminances in Youyang County and Qianjiang District were relatively low. The area with the lowest illuminance was not the area with the largest population density, which indicates that the factors affecting the annual illuminance are not only human production activities, but also may be related to geographical features such as the elevation and vegetation coverage. The terrain of Chongqing is gradually reduced from north to south to the Yangtze River Valley. In the northwest and middle parts of Chongqing, there are parallel mountain valleys with low mountains and hills. In the northeast, there are the Daba Mountains, and the Wuling Mountains are located in the southeast. The areas with the highest illuminance were located in the Yangtze River Valley with its low elevations, while the areas with the lowest illuminance were located in areas with high elevations. Therefore, elevation is an important factor affecting the illuminance on sunny days.

From Fig 9, it can be seen that the illuminance value of the central district in each season was lower than that of other areas in Chongqing, which was contrary to the distribution of aerosols; this trend was responsible for the inverse correlation between the AOD and illuminance in the model. The possible reasons are as follows.

First, the population density of the main city was large, and the emissions of aerosols from artificial sources were greater in this region, which led to the decreases in the illuminance. Second, in terms of topography, the hilly terrain in the southwest part of Chongqing was relatively low, but not conducive to the diffusion of pollutants. Because of the small population density and high elevations near the Yunnan-Guizhou Plateau, the northeast and southeast parts Chongqing were found to be relatively the most favorable regions for the daylight climate in Chongqing. It can be seen from the quarterly average hourly illuminance map that the high illuminance areas were the areas with high elevations and a low population density, while areas with low elevations and a high population density had low illuminances.

As shown in Fig 10, compared with the internal difference of each month, the clear day monthly average hourly illuminance difference between each month was more significant. In June, July, August, and September, the illuminance difference between regions in Chongqing was relatively small, while in October and November, the illuminance difference between regions in Chongqing was relatively large. July was the month with highest illuminance throughout the whole year. The clear day average monthly hourly illuminance of Chongqing was above 83,497 lx. December and January were the worst months, but the lowest illuminance was only 25,000 lx.

**Fig 11:**
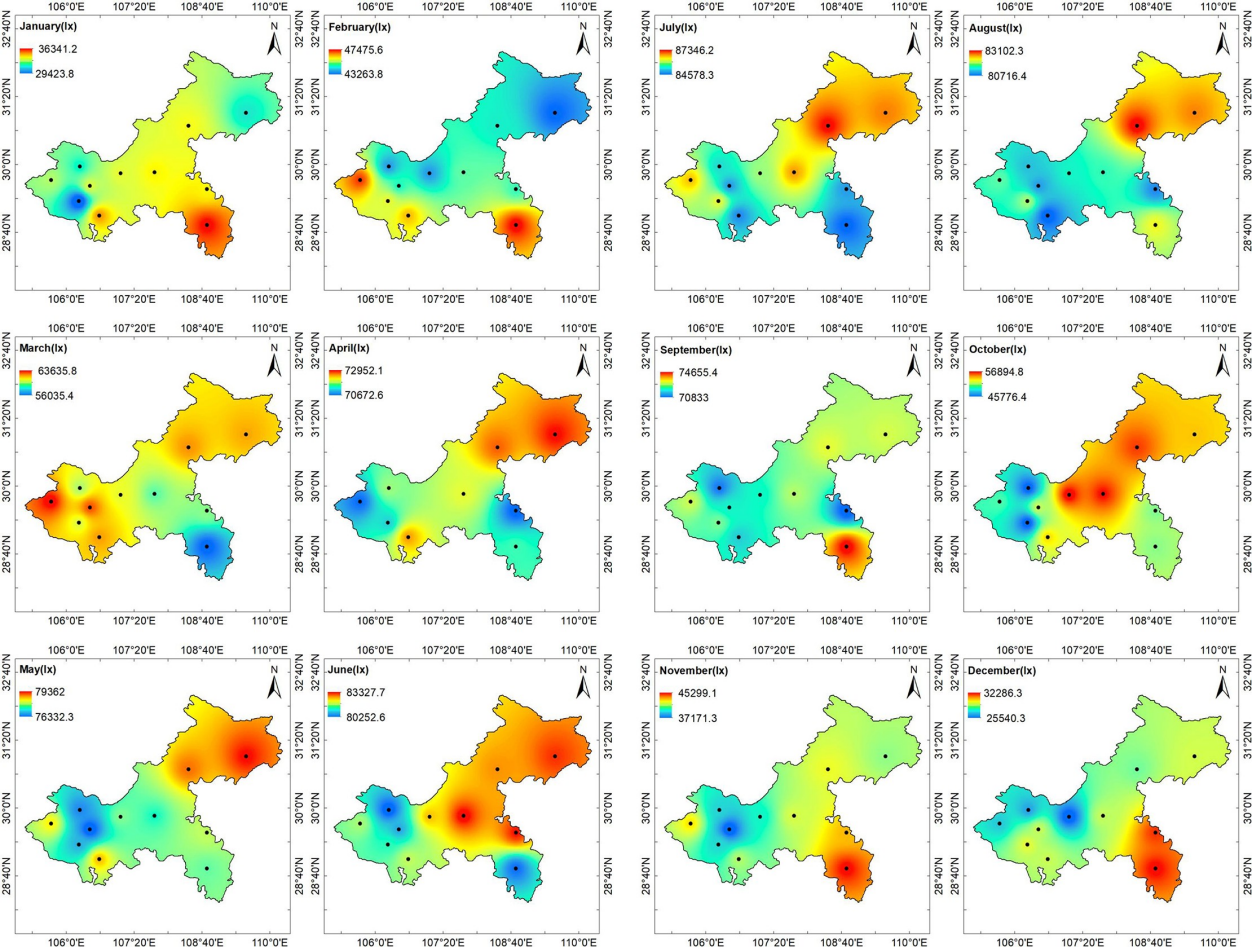
(a) Chongqing monthly average hourly illumination distribution with a different color scale at noon (from January to June). (b) Chongqing monthly average hourly illumination distribution with a different color scale at noon (from January to June).

To further understand the distribution of clear day illuminances in each month, based on Fig 10, different illuminance ranges were used to expand the color scale of each month, as shown in Fig 11. Fig 11 that the distribution of clear day illuminances in different months in the Chongqing area was very different. In January, the southeast part of Chongqing (Youyang County) had the highest illuminance value, while the Jiangjin District had a lower illuminance value. In February, the southeast part of Chongqing (Youyang County) also had the highest illumination value, while the lowest illumination value appeared in the northeast (Fengjie County). In March, the illumination value in the west (Dazu County) was the highest, while that in the southeast (Youyang County) was the lowest. In April, the highest illuminance was detected in the northeast (Fengjie County), while the lowest was detected in the east (Qianjiang District) and the west (Dazu County). In May, the highest illuminance was detected in the northeast (Fengjie County), while the lowest illuminance was detected in the main urban area (Shapingba). In June, the highest illuminances were detected in the northeast (Fengjie County, Wanxian) and the middle part of the study area (Fengdu County), while the lowest illuminances were detected in the central urban area and in the southeast (Youyang County). In July, the highest illuminance was detected in the middle part of the study area (Fengdu County), and the lowest illuminances were detected in the central urban area and in the southeast (Youyang County). The illuminance distribution in August was similar to that in July. In September, the highest illuminance was detected in the southeast (Youyang County), while the lowest illuminances were detected in the east (Qianjiang) and the main urban area (Hechuan District). In October, the highest illuminances were detected in the central area (Changshou District, Fengdu County, Wanxian), while the lowest illuminances were detected in the central urban area (Hechuan District, Jiangjin District). In November, the highest illuminance was detected in the southeast (Youyang County), while the lowest illuminance was detected in the central urban area. In December, the highest illuminance remained in the southeast (Youyang County), and the lowest illuminance was located around the central urban area (Changshou District, Hechuan District). From the above results, we concluded that the southeast high-elevation area has higher clear illuminance values than the other areas in autumn and winter, but the opposite trend appears in spring and autumn. At the same time, the illuminance value of the northeast Yangtze River Valley is higher in spring and summer, but lower in winter. In addition, clear illuminance in the main urban area is at a low value in each month, which may be related to the population density, pollution, and other factors.

## 4. Conclusion

In this paper, a clear day average hourly illuminance model was proposed. The solar altitude angle sine *sinh*, AOD *τ*, and atmospheric precipitable water content *w* were obtained from satellite data and ground measured data, while the regression coefficients were obtained from the regression analysis against measured illuminance data. This approach could resolve the problem of insufficient data for daylight design plans where ground measurements are not available. Clear day average hourly illuminance data obtained from this method agreed well with that obtained from ground measurements, with RMSD and MBD values of 22% and 0.05%, respectively. The model was then used to generate the spatial–temporal distribution maps of the clear day average hourly illuminance in Chongqing. The maps showed the annual, seasonal, and monthly variations of clear day average hourly illuminance in Chongqing. Although the model has been developed based on Chongqing data, it also could be applied for neighboring areas with a similar solar radiation climate such as in southwest China where the daylight data are insufficient.
